# Lung Functioning and Inflammation in a Mouse Model of Systemic Juvenile Idiopathic Arthritis

**DOI:** 10.3389/fimmu.2021.642778

**Published:** 2021-03-12

**Authors:** Bert Malengier-Devlies, Tatjana Decaesteker, Kaat Dekoster, Arno Vanstapel, Kourosh Ahmadzadeh, Fariba Poosti, Tania Mitera, Laura Seldeslachts, Erik Verbeken, Carine Wouters, Greetje Vande Velde, Jeroen Vanoirbeek, Patrick Matthys

**Affiliations:** ^1^Laboratory of Immunobiology, Department of Microbiology and Immunology, KU Leuven, Leuven, Belgium; ^2^Laboratory of Respiratory Diseases and Thoracic Surgery (BREATHE), Department of Chronic Diseases and Metabolism, KU Leuven, Leuven, Belgium; ^3^Biomedical MRI, Department of Imaging & Pathology, KU Leuven, Leuven, Belgium; ^4^Laboratory of Molecular Immunology, Department of Microbiology and Immunology, KU Leuven, Leuven, Belgium; ^5^Morphology and Molecular Pathology Section, University Hospitals Leuven, Leuven, Belgium; ^6^Division of Pediatric Rheumatology, University Hospitals Leuven, Leuven, Belgium; ^7^European Reference Network for Rare Immunodeficiency, Autoinflammatory and Autoimmune Diseases (RITA) at University Hospital Leuven, Leuven, Belgium

**Keywords:** lung inflammation, autoinflammation, mouse model, SJIA, IFN gamma

## Abstract

Systemic juvenile idiopathic arthritis (sJIA) is an immune disorder characterized by fever, skin rash, arthritis and splenomegaly. Recently, increasing number of sJIA patients were reported having lung disease. Here, we explored lung abnormalities in a mouse model for sJIA relying on injection of IFN-γ deficient (IFN-γ KO) mice with complete Freund's adjuvant (CFA). Monitoring of lung changes during development of sJIA using microcomputer tomography revealed a moderate enlargement of lungs, a decrease in aerated and increase in non-aerated lung density. When lung function and airway reactivity to methacholine was assessed, gender differences were seen. While male mice showed an increased tissue hysteresivity, female animals were characterized by an increased airway hyperactivity, mirroring ongoing inflammation. Histologically, lungs of sJIA-like mice showed subpleural and parenchymal cellular infiltrates and formation of small granulomas. Flow cytometric analysis identified immature and mature neutrophils, and activated macrophages as major cell infiltrates. Lung inflammation in sJIA-like mice was accompanied by augmented expression of IL-1β and IL-6, two target cytokines in the treatment of sJIA. The increased expression of granulocyte colony stimulating factor, a potent inducer of granulopoiesis, in lungs of mice was striking considering the observed neutrophilia in patients. We conclude that development of sJIA in a mouse model is associated with lung inflammation which is distinct to the lung manifestations seen in sJIA patients. Our observations however underscore the importance of monitoring lung disease during systemic inflammation and the model provides a tool to explore the underlying mechanism of lung pathology in an autoinflammatory disease context.

## Introduction

Systemic juvenile idiopathic arthritis (sJIA) is a childhood auto-inflammatory immune disorder, characterized by growth retardation, spiking fever, skin rash, arthritis, splenomegaly and polyserositis. Hematological changes, such as leucocytosis, neutrophilia, anemia and thrombocytosis are typically seen. High levels of interleukin-6 (IL-6), IL-18, C-reactive protein (CRP) and S100 acute phase proteins are present in plasma of sJIA patients (1–6). Around 10–15% of patients develop a live-threatening complication, called macrophage activation syndrome (MAS) which is associated with pancytopenia, increased liver enzymes, high ferritin levels, and coagulopathy (1, 7–9). MAS is associated with high production of IL-18 and interferon gamma (IFN-γ) (10). Part of sJIA patients can successfully be treated with biologics targeting IL-1β or IL-6, which replaced chronic high dose treatment with corticosteroids (11–14).

Chronic parenchymal lung disease (PLD) has only rarely been reported in sJIA (15, 16). Kimura Y *et al* described for the first time 25 sJIA patients with chronic pulmonary disease, characterized by pulmonary arterial hypertension (PAH), interstitial lung disease (ILD) and pulmonary alveolar proteinosis (PAP) (17). Recently, two studies reported lung disease in patients with sJIA (18, 19). Radiographic analysis demonstrated ground-glass opacities, subpleural reticulation, interlobular septal thickening and lymphadenopathy. Histopathological analysis revealed lymphoplasmacytic infiltration, mixed features of PAP or endogenous lipoid pneumonia (ELP), vasculopathy, alveolar epithelial cell hyperplasia and collagenous fibrosis. Although sJIA equally occurs in both sexes, a non-significant higher incidence of female patients with lung disease (66%) was reported (19). The incidence of lung disease cases has increased since 2010 and coincides with the increasing use of biologics, Nigrovic *et al*., highlighted that “lung disease in sJIA might be a dark cloud on the horizon in the management of sJIA” (20, 21). Indeed, treatment with IL-1 or IL-6 inhibitors was demonstrated as a risk factor in the development of sJIA lung disease (19).

In this study, we investigated lung pathology in a mouse model for sJIA. The model relies on a single injection of BALB/C mice with complete Freund's adjuvant (CFA). While wild-type (WT) mice develop a transient and mild form of inflammation, IFN-γ-deficient (IFN-γ KO) mice develop a chronic inflammatory condition with clinical, hematological and biological characteristics reminiscent to sJIA (22). Although sJIA patients do not have mutations in the IFN-γ gene, defects in the production of IFN-γ by NK cells have been reported (23, 24). Furthermore, the clinical relevance of the model lies in the many similarities with sJIA, including weight loss, arthritis, skin rash, splenomegaly, lymphadenopathy, anemia, granulocytosis, thrombocytosis, NK cell defects and increased serum levels of IL-6 (Supplementary Table 1). To study lung inflammation, we evaluated the functional, histopathological and cellular aspects of lungs from CFA-injected IFN-γ KO mice (also designated as sJIA-like mice) and included CFA-injected WT mice for comparison.

## Materials and Methods

### Mice Immunization

IFN-γ KO and WT BALB/c mice were bred under specific pathogen-free conditions. Six-to 8-week-old mice were either or not subcutaneously injected at the tail base with complete freund's adjuvant (CFA) (Difco) containing heat-killed *Mycobacterium butyricum* (1.5 mg/ml) as described (Supplementary Table 1) (22). All animals experiments were approved by the Ethics Committee of KU Leuven (P182/2014).

### Micro-Computed Tomography

Micro-computed tomography was performed on day 0, day 12, and day 21 post CFA-immunization. During the procedure, the mice were anesthetized by inhalation of 1.5–2% isoflurane in 100% oxygen and scanned in supine position using a dedicated small animal *in vivo* μCT scanner (Skyscan 1278, Bruker μCT, Kontich, Belgium) as previously described (25). The following parameters were used: 50 kVp X-ray source voltage and 346 μA current combined with a composite X-ray filter of 1 mm aluminum, 150 ms exposure time per projection, acquiring three projections per step with 0.9° increments over a total angle of 220°, and 10 cm field of view covering the whole body producing expiratory-weighted 3D data with 50 μm isotropic reconstructed voxel size. Each scan takes approximately 3 min and is associated with a measured radiation dose of 60–80 mGy (26). Software provided by the manufacturer (NRecon, DataViewer, and CTan) was used to reconstruct, visualize, and process μCT data as described previously (25–27). For Hounsfield unit (HU) calibration, a phantom was scanned consisting of an air-filled 1.5 ml tube inside a water-filled 50 ml tube. Based on full stack histograms of a volume-of-interest (VOI) containing only water or air, the main grayscale index of water (127) was set at 0 HU and grayscale index of air (6) at −1000 HU. The non-aerated lung volume, aerated lung volume, total lung volume and respective densities within these volumes were quantified for a manually delineated VOI covering the lung, avoiding the heart and main blood vessels. The aerated and non-aerated lung densities represents the density of air in the alveoli and the surrounding epithelial, capillary and extracellular matrix and small airways respectively. Oedema and accumulation of inflammatory cells are associated with an increased density whereas destruction of the alveoli would result in a decreased lung density. The threshold used to distinguish aerated from non-aerated lung volume was manually set at −180 HU and kept constant for all data sets.

### Lung Function Measurements

At day 21 post CFA-infection, on the moment all overt signs of inflammation were discernible, lung function measurements were performed and mice were subsequently euthanized. During function measurements, mice were anesthetized by an intraperitoneal (i.p.) injection of pentobarbital (70 mg/kg body weight). Lung function measurements were performed using a forced oscillation technique (FlexiVent 7.6, SCIREQ, Montreal, Canada), as described previously (28). Mice were quasi-sinusoidally ventilated with a tidal volume of 10 mL/kg at a frequency of 150 breaths/min and a positive end-expiratory pressure of 3 cm H_2_O, to mimic the characteristics of spontaneous breathing. Airway resistance (Rn) was measured using the “quick-prime 3” protocol, which induces oscillations of 1 to 20.5 Hz during 3 s. Lung volumes, such as forced expiratory volume in 0.1s (FEV_0.1_), forced vital capacity (FVC) and peak expiratory flow (PEF) were measured by using the ‘Negative Pressure Forced Oscillation’ technique. After baseline measurements, each mouse was exposed to a methacholine aerosol, generated with an in-line nebulizer (Aeroneb Lab nebuliser, 2.5–4 μm, Aerogen, Galway, Ireland) and administered at increasing concentrations (0, 2.5, 5, 10, 20 and 40 mg/ml), each during 5 s, and Rn measured.

### Histopathology and Immunofluorescence Staining

Lungs were fixed for 24 h with 10% formalin and were kept inflated at constant pressure throughout the fixation process. Lung sections were stained with hematoxylin and eosin, and were evaluated blindly by two experienced lung pathologists. Images were taken with a Leica DFC295 microscope. Immunofluorescence staining was performed on 4 μm acetone-fixed frozen lung and kidney sections. Tissue sections were incubated with the primary anti-α-SMA (Cat. Nr: 61001, ProGen, Heidelberg, Germany) antibody for 1 h at RT. Binding of α-SMA primary antibody was detected by incubation with goat anti-mouse IgG2a FITC (Southern Biotech, Birmingham, AL, USA) for 30 min. Nuclei were stained with Hoechst, and sections were coverslipped with Prolong Gold antifade reagent (Invitrogen). All immunofluorescence images were taken with a Zeiss Axiovert 200M inverted microscope and AxioVision acquisition software (Carl Zeiss, Oberkochen, Germany).

### Antibodies, Flow Cytometry and FACS Sorting

To prepare single cell suspensions, lungs were digested for 45 min at 37°C in RPMI 1640 supplemented with 5 % FCS, 2 mM L-glutamine, 0.05 mM 2-mercaptoethanol, 100 U/ml penicillin, 100 mg/ml streptomycin (Invitrogen), 1 mg/ml collagenase type 2 (Worthington Biochemical), and 0.02 mg/ml DNase I (grade II from bovine pancreas, Ingelheim). Red blood cells were lysed using ACK Lysing Buffer (Gibco). Cells were incubated with FcR-block (Miltenyi Biotec) and stained extracellularly in FACS buffer (PBS +2% FCS +2mM EDTA). Antibodies used in this study are listed in Supplementary Table 2. Before lineage gating, all populations were first gated on live cells by using Zombie Aqua 516 (Biolegend). Forward and side scatter was used to limit debris and doublets. Cells were run on a BD LSRFortessa X20 or a BD FACSymphony equipped with the DIVA software and data were analyzed using FlowJo software (LLC, V10).

### qRT-PCR

Total RNA was extracted by PureLink RNA Mini kit (Invitrogen) or the RNeasy Micro kit (Qiagen), followed by the preparation of complementary DNA (cDNA) using Superscript II reverse transcriptase and random primers (Invitrogen). Real-time (RT) quantitative (q)PCR was performed using a TaqMan gene expression assay (Applied Biosystems) on a 7500 Real-Time PCR System Apparatus. Primers used are listed in Supplementary Table 3. All primers were obtained from Integrated DNA Technologies and RNA expression was normalized to the expression of GAPDH RNA (Mm99999915_g1) according to the delta-delta CT method.

### Statistical Analyses

The two-tailed non-parametric Mann-Whitney U test was used for all single comparisons of two unpaired groups. For data with three or more groups, the non-parametric Kruskal-Wallis test (unpaired data) or Friedman test (paired data) was used and was followed by a Mann-Whitney *U* test. Data of longitudinal μCT-derived biomarkers (mean lung density, total lung volume, non-aerated lung volume and aerated lung volume) were analyzed using a mixed-effects model with Geisser-Greenhouse correction. For multiple comparison, Sidak's *post hoc* test was used. For correlation analysis, the Spearman correlation coefficient was determined. For all tests, statistical significance was assumed with ^*^p-value < 0.05; ^**^p-value < 0.01; ^***^p-value < 0.001; ^****^p-value < 0.0001. All data analysis was performed using the GraphPad Prism 7 software.

## Results

### Changes in Lung Volume and Lung Density Upon Immunization With CFA

**Figure 1:**
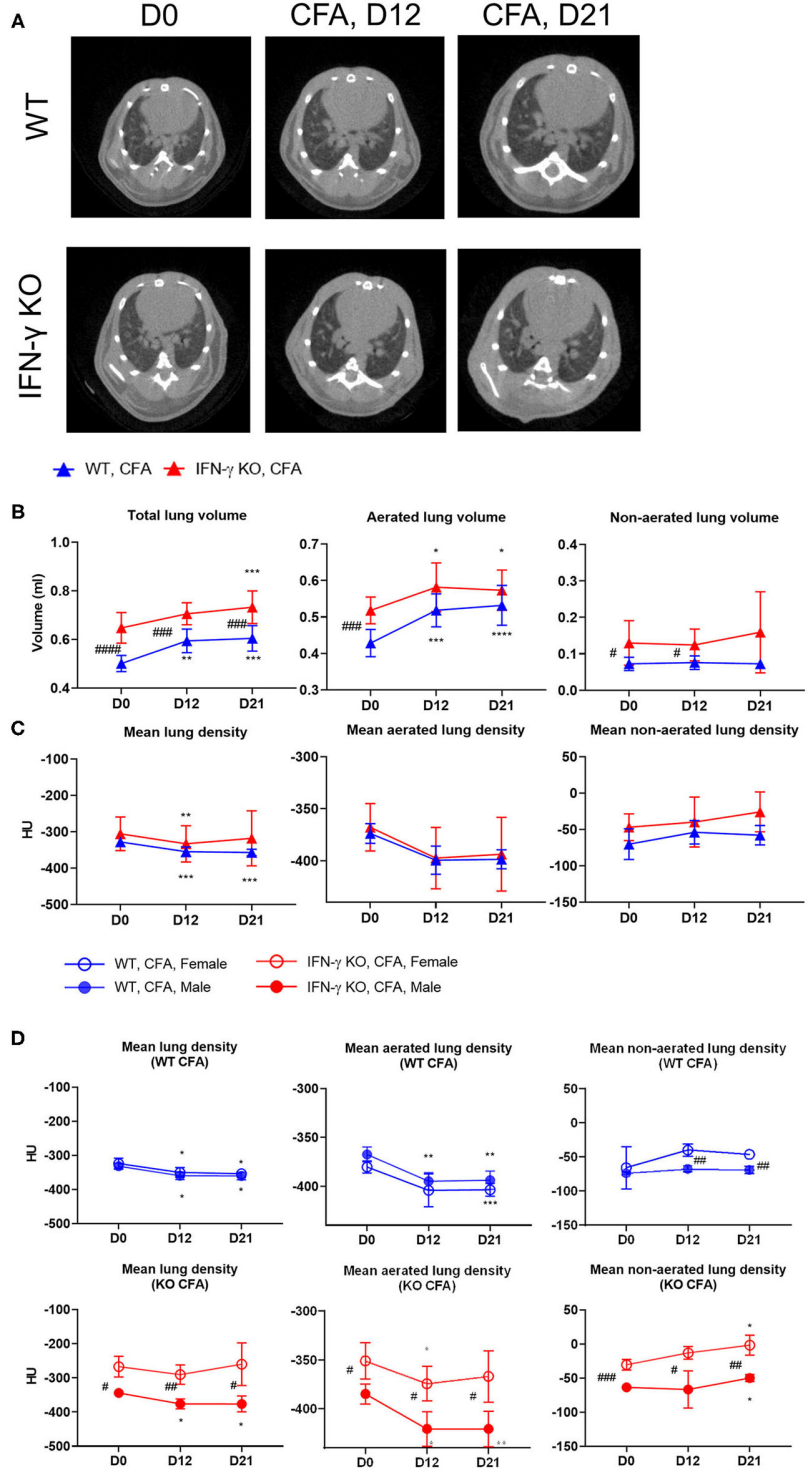
WT mice and IFN-γ KO mice were immunized with CFA. Mice were scanned with μCT before (day D0) and at regular time points after the CFA-immunization (day D12, day D21). **(A)** Representative transverse μCT images of a WT and KO CFA-immunized mice at the selected time points. The position of the shown images was choses at 100 z-positions after the first bifurcation of the airways. **(B)** Three lung volumes were established: total lung volume (ml), aerated long volume (ml) and non-aerated long volume (ml). **(C,D)** The mean lung density, aerated lung density and non-aerated lung density were measured and expressed in HU units. Data are represented as mean group values with standard error (SE). Blue graphs represent CFA-immunized WT mice, red graphs represent CFA-immunized IFN-γ KO mice. ^*^/# *p* < 0.05; ^**^/## *p* < 0.01; ^***^/### *p* < 0.001; ^****^/#### *p* < 0.0001 by repeated measures two-way ANOVA. Asterisk represents time-based differences and hashtag represents differences between WT and IFN-γ KO mice.

When BALB/C mice are injected s.c. at the base of the tail with CFA, they develop systemic inflammation within 21 days. While the inflammation is mild in WT animals, IFN-γ KO mice develop a more severe disease with symptoms and blood abnormalities that are similar to those reported in sJIA patients (Supplementary Table 1) (22). To identify potential lung disease during development of systemic inflammation, mice were monitored by high-resolution low-dose *in vivo* μCT before and after immunization with CFA, that is, at day 12 when sJIA symptoms started to appear and at day 21 when blood abnormalities are maximal in both groups of mice (22). Representation of μCT images and quantification of lung volume and lung densities are shown in Figure 1 and Supplementary Figure 1. Visual inspection of the μCT images showed a moderate enlargement and a reduced density of the lungs over time in CFA-immunized mice (Figure 1A). Surprisingly, IFN-γ KO mice have a larger lung volume than WT mice, even before the start of immunization despite the fact that the mean body weight in both groups was similar at day 0 (Figure 1B, body weights are not shown). Following CFA-immunization, total lung volume increased in both WT and IFN-γ KO mice which was attributed to an increase in aerated lung volume. CFA-challenge also resulted in a decrease of mean lung density in both WT and IFN-γ KO mice (Figure 1B). While total and aerated lung densities decreased upon immunization in both strains, the non-aerated density significantly increased in IFN-γ KO mice only (Figure 1C). When aerated and non-aerated lung densities were plotted, the changes reached statistical significance when data were separately quantified in females and males (Figure 1D). In conclusion, development of systemic inflammation elicited by CFA, is associate with a moderate increase in aerated lung volume and a decrease in aerated lung density, the latter being indicative for loss of alveolar integrity. The more severe inflammation in IFN-γ KO mice is associated with an increased non-aerated lung density, which may be indicative for inflammatory infiltrates (25).

### Functional Lung Alterations in CFA-Challenged IFN-γ KO Mice

**Figure 2:**
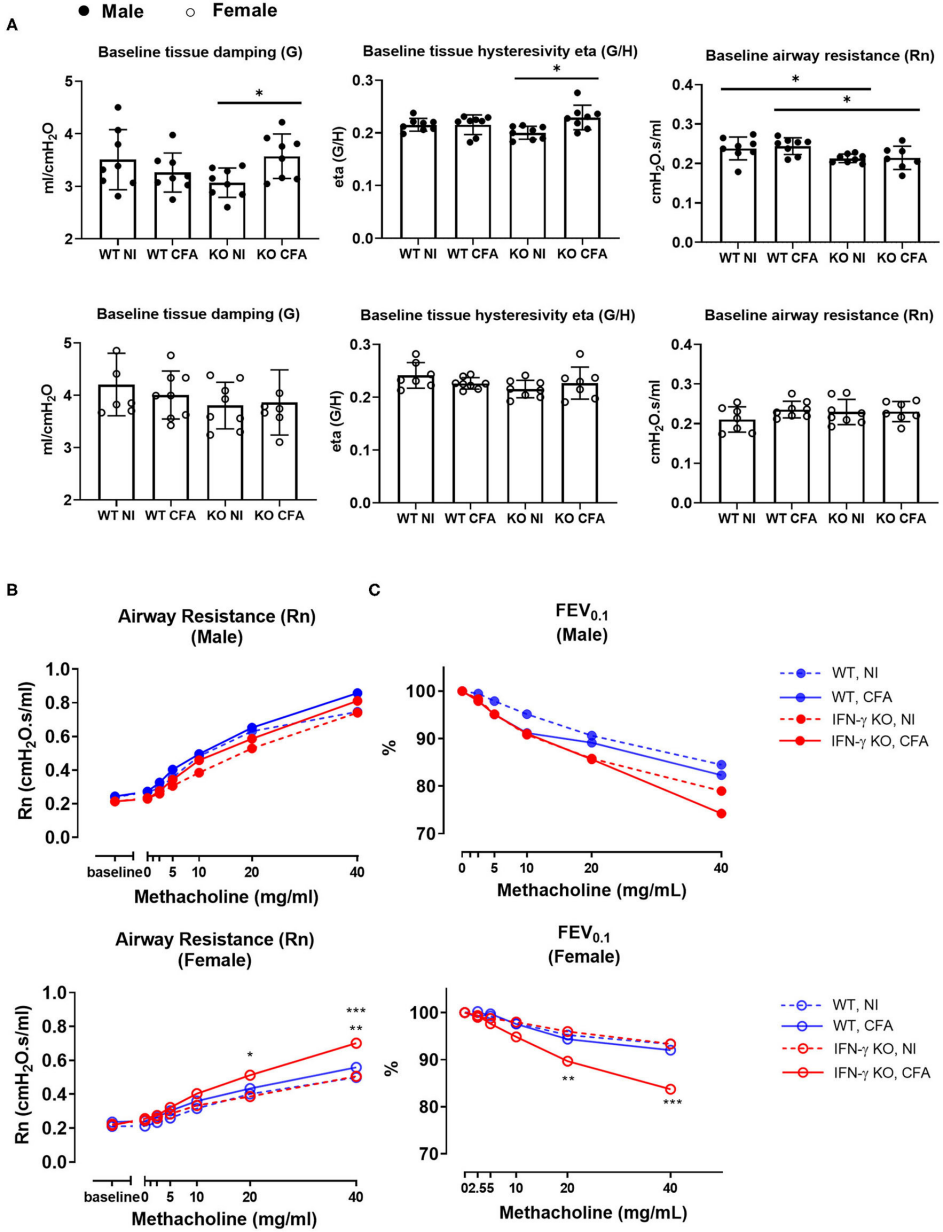
WT mice and IFN-γ KO mice were immunized with CFA. NI WT and IFN-γ KO mice were included as controls. Baseline lung function parameters and airway hyperreactivity were assessed at day 21 post CFA-injection. **(A)** Baseline tissue damping **(G)**, tissue hysteresivity (eta = G/H) and airway resistance (Rn) were measured using a forced oscillation technique (QP3) in both male (closed) and female (open) mice. Data are represented as mean group values and individual data points. *p < 0.05 by by Kruskal-Wallis followed by Mann-Whitney U test. **(B)** The dose-response of the airway resistance to methacholine (0–40 mg/ml), in both male (closed) and female (open) mice, was measured using the forced oscillations technique (QP3). *p < 0.05 for IFN-γ KO CFA compared to IFN-γ KO NI at 20 mg/ml. **p < 0.01 and ***p < 0.001 for IFN-γ KO CFA compared to WT CFA and IFN-γ KO NI respectively at 40 mg/ml (Two-Way ANOVA). **(C)** The dose-response of the forced expiration volume in 0.1 sec (FEV0.1) to methacholine (0–40 mg/ml) was measured using a negative pressure forced expiration maneuver (NPFE), in male (closed) and female (open) mice. **p < 0.01 for IFN-γ KO CFA compared to IFN-γ KO NI at 20 mg/ml. ***p < 0.001 for all groups compared to IFN-γ KO CFA at 40 mg/ml. (Two-Way ANOVA). Blue graphs represent CFA-immunized WT mice, red graphs represent CFA-immunized IFN-γ KO mice. Figures 2B,C show the mean values of Rn and FEV0.1, respectively, against the methacholine concentrations between 0 and 40 mg/ml. (*n* = 7–8).

On day 21, a time-point on which most systemic features were apparent (22), lung function and non-specific airway reactivity to methacholine was assessed. Distinct functional alterations were measured between male and female mice, therefore both sexes are shown separately. Lung forced vital capacity (FVC), forced expiratory volume 0.1 (FEV_0.1_) and the FVC/FEV_0.1_ ratio (also known as Tiffeneau-index 0.1) did not show any difference between the experimental groups (data not shown). In contrast, lung structural parameters, such as tissue damping (G) and tissue hysteresivity (eta=G/H) were significantly higher in male CFA-challenged IFN-γ KO mice compared to naïve IFN-γ KO mice. A significant lower airway resistance (Rn) in male IFN-γ KO mice compared with male WT mice, regardless of the CFA injection was also observed. These changes in G, eta and Rn were not seen in female mice (Figure 2A). After the baseline measurements, a dose-response challenge with methacholine was performed to evaluate the airway hyperreactivity. Airway resistance of female CFA-challenged IFN-γ KO mice was significantly higher at 20 and 40 mg/ml compared with other treatment groups (Figure 2B). This was also reflected in a significantly lower FEV_0.1_ upon methacholine challenge with 20 and 40 mg/ml in female CFA-challenged IFN-γ KO mice (Figure 2C), confirming airway hyperreactivity in female CFA-challenged IFN-γ KO mice. In male mice, all dose-responses to methacholine were the same and no airway hyperreactivity could be measured (Figure 2B, C). In conclusion, CFA-immunization leads to functional lung alterations in IFN-γ KO mice. Whereas male mice show increased tissue hysteresivity, female mice were characterized by an increased airway hyperreactivity.

### Histopathology Reveals Cell Infiltration and Granulomas in Lungs of CFA-Challenged Mice

**Figure 3:**
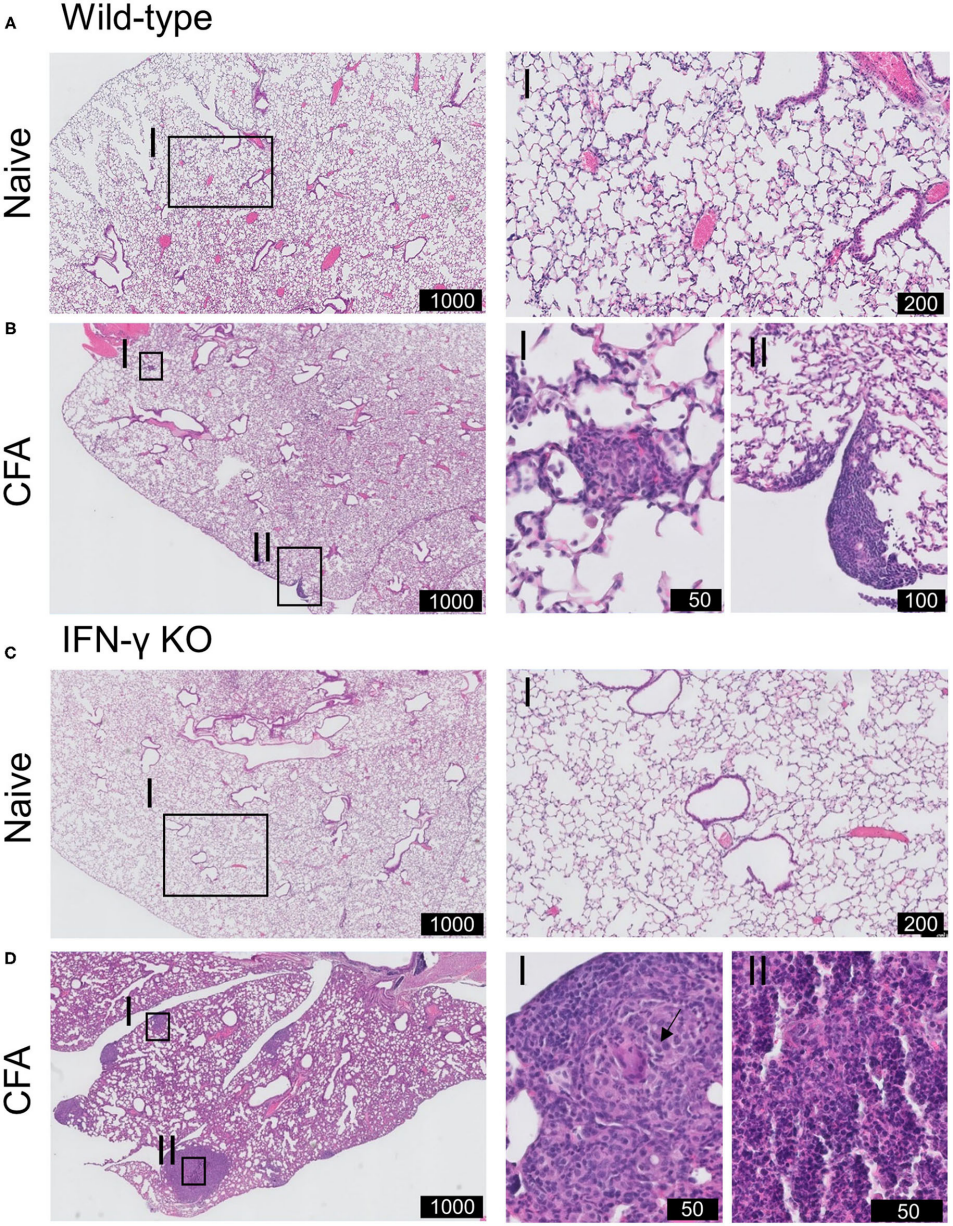
Histopathology of pulmonary involvement in CFA-immunized mice. WT and IFN-γ KO mice were immunized with CFA. NI WT and IFN-γ KO mice were include as controls. **(A)** Representative H&E-stained lung section of WT mice illustrating the normally preserved parenchyma. I is enlargement of the delineated area. **(B)** Representative H&E-stained lung section of CFA-challenged WT mice at day 21. I and II highlights the presence of subpleural nodules. The parenchyma is further normally preserved. **(C)** Representative H&E-stained lung section of KO mice illustrating the normally preserved parenchyma. I is enlargement of the delineated area. **(D)** Representative H&E-stained lung sections of CFA-challenged IFN-γ KO mice at day 21. I, II are enlargements of the delineated areas. Scale is indicated in the black boxes (in μm). Arrow indicate multinucleated giant cells in granuloma-like cells lesions.

On day 21 post-immunization, lungs of mice were histopathologically evaluated (Figure 3). Lungs of naïve WT and IFN-γ KO mice had a normal preserved parenchymal appearance without cellular infiltrates. In contrast, histological alterations were observed in both CFA-immunized WT and IFN-γ KO mice. Subpleural and parenchymal cellular infiltrates were present, ranging from small nodular lesions comprised of macrophages and lymphocytes to overt granulomatous inflammation, comprised of prominent multinucleated giant cells and mainly lymphocytic inflammation. No granulomas were reported in other organs including liver, kidney, brain or colon (22). Granulomas in IFN-γ KO mice were more extensively present and also contained scattered neutrophils. No necrosis nor neutrophilic inflammation was found in immunized WT mice. Furthermore, CFA-challenged IFN-γ KO mice showed a mild diffuse interstitial lymphocytic inflammation. In some of the immunized mice, widening of the alveolar spaces was observed, consistent with the observed decrease in aerated lung density. However, no intra-alveolar pathology was present and there was no evidence of PAP or oedema. No overt signs of fibrosis were observed and the absence of collagen deposition was confirmed by an immunohistochemical staining of Alpha-smooth muscle actin (α-SMA) (Supplementary Figure 2). In conclusion, a single injection of CFA at the base of the tail leads to a predominantly monocyte, macrophage and neutrophilic pulmonary infiltration in both WT and IFN-γ KO mice which was more pronounced in the sJIA-like animals.

### Lungs of CFA-Immunized IFN-γ KO Mice Present With Elevated Numbers of Neutrophils and Activated Monocytes/Macrophages

**Figure 4:**
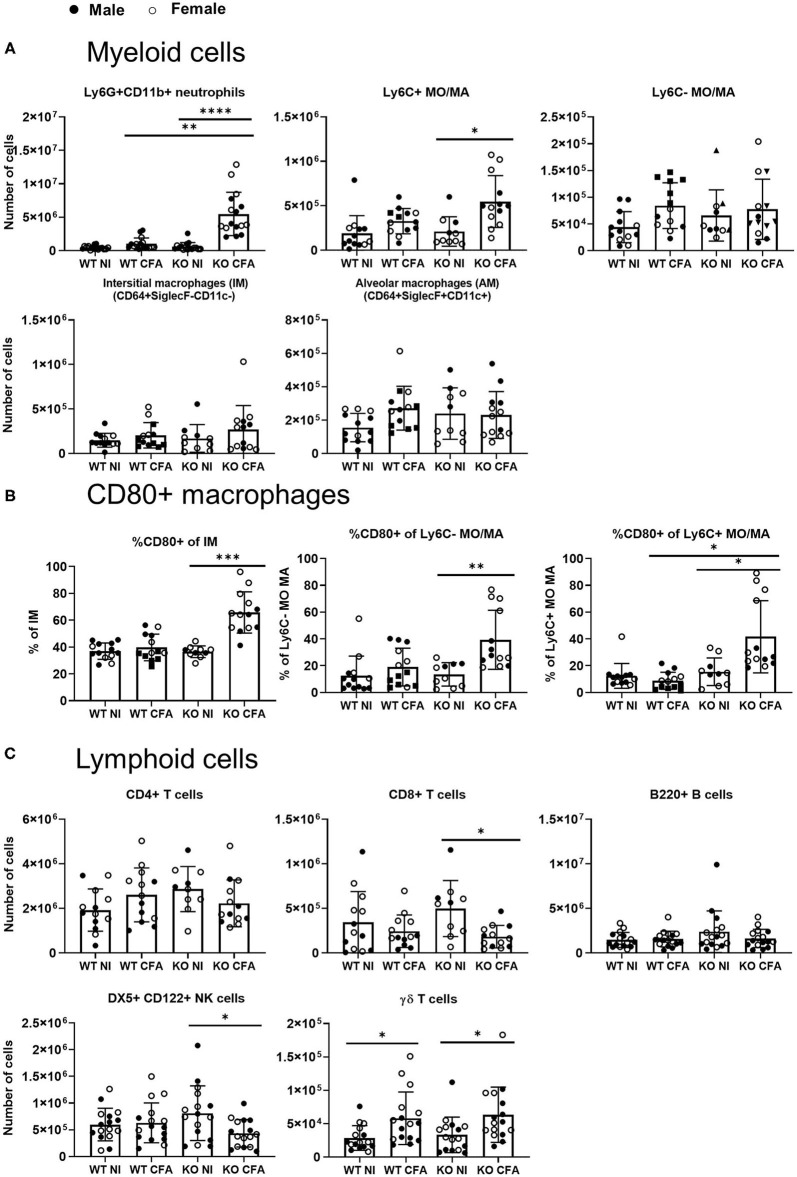
WT mice and IFN-γ KO mice were immunized with CFA. NI WT and IFN-γ KO mice were included as controls. Lung cells were analyzed by flow cytometry. **(A)** Absolute number of Ly6G+CD11b+ neutrophils, CD11b+MHCII-Ly6C_pos_ monocytes/macrophages (MO/MA), CD11b+MHCII-Ly6C_neg_ MO/MA, CD64+SiglecF-CD11c- interstitial macrophages (IM) and CD64+SiglecF+CD11c+ alveolar macrophages (AM) were measured. **(B)** Percentage of CD80+, were determined within the interstitial macrophages (IM), Ly6C- MO/MA and the Ly6C+ MO/MA. **(C)** Absolute number of CD4+ T cells, CD8+ T cells, B220+ B cells, DX5+CD122+ NK cells and CD3+γδTCR+ γδ T cells were measured. Dots represent individual mice (male closed, female open), horizontal bars represent mean group values with standard error (SE). *p < 0.05; **p < 0.01; ****p < 0.0001 by Kruskal-Wallis followed by Mann-Whitney *U* test.

To further characterize the observed pulmonary inflammation, flow cytometric analysis was applied on single cell suspensions of lung tissues. As can be seen in Figure 4A, numbers of neutrophils were significantly increased in CFA-IFN-γ KO mice. In sJIA patients with lung disease, it was hypothesized that the inflammatory milieu could polarize macrophage away from the phenotype necessary for recycling the surfactant (18). We therefore characterized four different subsets of monocytes/macrophages (MO/MA) in lung of our mice, more specifically classical inflammatory Ly6C^+^ MO/MA, non-classical patrolling Ly6C^−^ MO/MA, interstitial macrophages (IM) and alveolar macrophages (AM) (29). As shown in Figure 4A, the Ly6C+ MO/MA subset was found to be significantly increased in CFA-challenged KO mice, while other subsets remained unchanged. Interestingly, when CD80 positive macrophages were determined, which is considered as an activating marker and M1-like marker (30), CFA-immunized IFN-γ KO mice showed a significantly higher percentage of CD80^+^ Ly6C^+^ MO/MA, Ly6C^−^ MO/MA and IM (Figure 4B). Numbers of SiglecF+CD64- eosinophils, CD11b+ dendritic cells (DC) and CD103+ DCs were increased upon CFA-immunization in WT mice, whereas they were only slightly elevated in immunized IFN-γ KO mice (Supplementary Figures 3A–C).

Number of lymphocytes were either unchanged (CD4+ T cells or B220+ B cells) or were significantly decreased in the diseased IFN-γ KO mice (CD8+ T cells and NK cells). In analogy with our previous data obtained in spleen and LNs (22), CFA-immunization was associated with an increased number of γδ T cells in lungs of both WT and IFN-γ KO mice (Figure 4C). In all studied leukocyte populations, no differences were found between male and female mice. Nevertheless, the highest monocyte/macrophage activation was seen in the female mice. In conclusion, myeloid cell number and activation of macrophages are elevated in the lung tissues of CFA-immunized IFN-γ KO mice.

### CFA-Immunized IFN-γ KO Mice Show Increased Expression of Inflammatory Mediators in Lung Tissue

**Figure 5:**
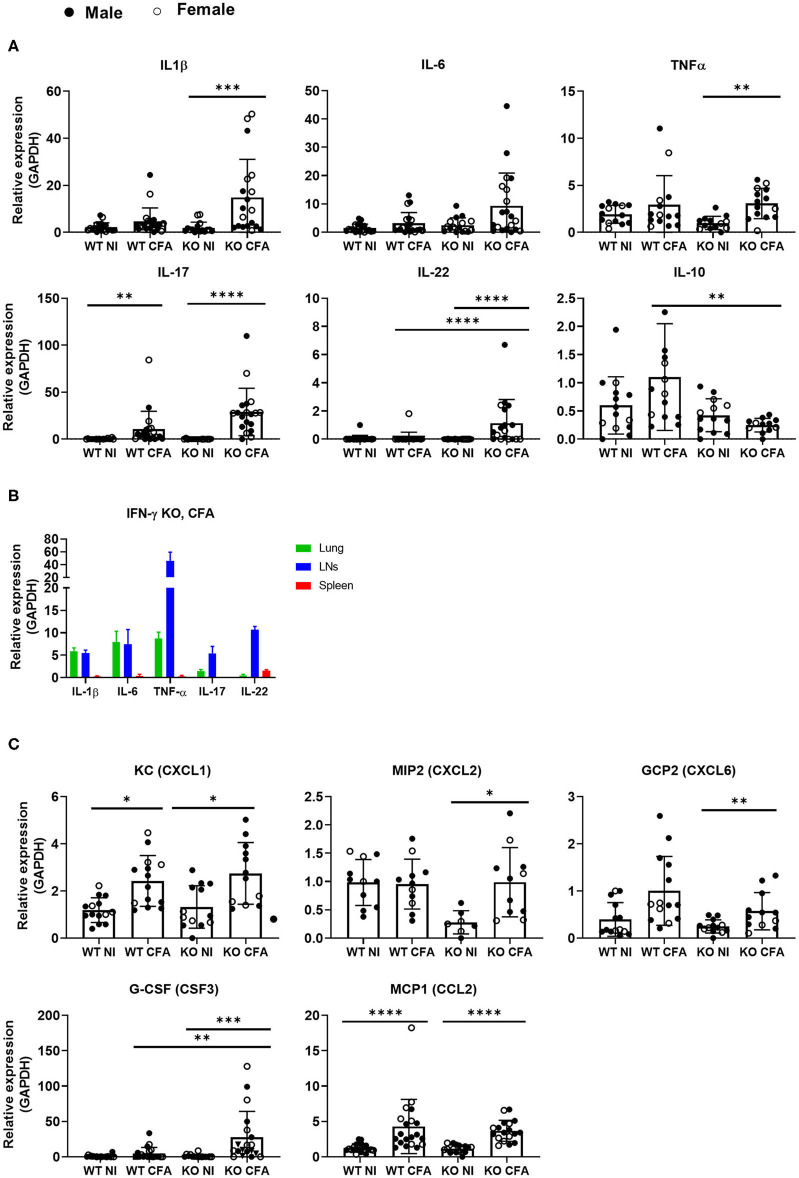
WT mice and IFN-γ KO mice were immunized with CFA. NI WT and IFN-γ KO mice were included as controls. **(A)** The relative RNA expression of IL-1β,IL-6, TNFα, IL-17, IL-22, and IL-10 was measured in the lungs. **(B)** Expression levels of IL-1β, IL-6, TNF-α, IL-17 and IL-22 were compaed between lungs, lymph nodes and spleens. **(C)** Relative RNA expression of the main chemokines or growth factors: KC (CXCL1), MIP2 (CXCL2), GCP2 (CXCL6), G-CSF (CSF3) and MCP1 (CCL2) were determined in the lungs by qPCR. Dots represent individual mice (female open, male closed), horizontal bars represent mean group values with standard error (SE). *p < 0.05; **p < 0.01; ***p < 0.001; ****p < 0.0001 by Kruskal-Wallis followed by Mann-Whitney *U* test.

To assess the inflammatory mediators involved in the observed lung inflammation, mRNA levels of inflammatory cytokines and chemokines were measured by qPCR in lung tissue of the four groups of mice (Figure 5). Upon CFA-immunization, expression of the pro-inflammatory cytokines IL-1β, IL-6, TNFα, IL-17, and IL-22 increased significantly in predominantly in IFN-γ KO mice. IL-10, an anti-inflammatory cytokine was significantly lower in CFA-challenged IFN-γ KO vs. WT mice (Figure 5A). In a replicate experiment, cytokines were measured in the lungs, lymph nodes and spleens of CFA-immunized IFN-γ KO mice. Intriguingly, levels of IL-1β and IL-6 in lung tissue were similar or higher than levels found in lymph nodes and spleen respectively, pointing to the importance of a non-lympoid organ as source for these two cytokines (Figure 5B). Analysis of the type I and the type II IFN genes, exposed an increased expression of CXCL9 and CXCL10 in CFA-immunized WT mice only (Supplementary Figure 4A). Since neutrophils and monocyte/macrophages were the most abundant infiltrating cells in lung tissue of CFA-challenged mice, we examined expression of the main neutrophil and monocyte/macrophage chemoattractants: keratinocyte chemoattractant (KC) (CXCL1), macrophage inflammatory protein 2 (MIP2 / CXCL2), granulocyte chemotactic protein 2 (GCP2/CXLC6) and monocyte chemoattractant protein 1 (MCP1). All of the chemokines were significantly increased in immunized IFN-γ KO mice and two in WT counterparts. We further analyzed granulocyte colony stimulating factor (G-CSF), a potent inducer of granulopoiesis, and found significant increased expression levels in lung tissue of CFA-induced IFN-γ KO mice, as compared to its naïve and WT counterparts, and these data may explain the observed neutrophilia in these mice (Figure 5C). Furthermore, a positive correlation was found between the number of neutrophils or the number of conventional inflammatory Ly6C_pos_ monocytes and the relative expression of either IL-1β, IL-17, G-CSF or MCP-1. In addition, a positive correlation could be made between the absolute number of neutrophils and the expression levels of IL-22 (Supplementary Figures 4B,C). From these results, we conclude that lungs of sJIA-like mice are represent a relevant basis of neutrophil and MO/MA-associated inflammatory mediators.

## Discussion

Pleuritis and pleural effusions are commonly seen in sJIA patients whereas PLD was rarely described until 2015 (15–17). Considering the increased reports on pulmonary manifestations in patients with sJIA (18, 19), we investigated the physiological and potential inflammatory aspects of lungs in a CFA-based mouse model of sJIA. We found that following immunization, mice display altered lung physiological responses and develop pulmonary inflammation with cellular infiltration and expression of pro-inflammatory cytokines. In comparison with CFA-challenged WT mice, IFN-γ KO mice showed a distinct and more severe pathology, which is in line with their increased expression of clinical, hematological and biological sJIA-like features.

Non-invasive μCT analysis was able to detect mild changes during the progression of the disease, including increases in lung volume and changes in lung density. The increased lung volume was mainly due to an increase in the aerated lung volume which is a phenomenon that has previously been described in mice having lung infections, inflammation or fibrosis, and which is absent in humans (25). This compensatory increase in the aerated lung volume may underestimate the presence of a restrictive lung disease during lung function volumetric readouts. In our study, lung function measurements showed indeed no difference in forced vital capacity. The overall or aerated lung density was decreased upon immunization with CFA, without clear-cut differences between IFN-γ KO and WT mice. The gradual changes in aerated lung volume or density could reflect longitudinal changes measured in healthy adult mice but are probably neglectable since the short duration of the experiment (25). When lungs were subjected to functional measurements, subtle differences were found in IFN-γ KO mice, provided that females and males were analyzed separately. The induction of sJIA-like disease in IFN-γ KO mice was associated with significantly increased long tissue damping and hysteresivity, in male mice and with an increased airway hyperreactivity in females. Although the observed functional changes in the lungs may be related to the increased infiltration of immune cells in the lungs of IFN-γ KO mice compared to WT counterparts, it needs to be said that no gender differences were observed in the flow cytometric and histological analysis. Note that also in sJIA patients, no gender differences were described. Histologically, the lungs of CFA-immunized mice (both WT and IFN-γ KO animals) showed subpleural and parenchyma cellular infiltrates with formation of granulomas. More extensive granulomas were found in IFN-γ KO mice, in line with the increased non-aerated lung consolidation on μCT. Flow cytometric analysis of lung tissue homogenates revealed significantly elevated numbers of neutrophils and Ly6C_pos_ monocyte/macrophages in lungs of CFA-immunized IFN-γ KO mice, while numbers of cytotoxic T cells and NK cells were lower. γδ T cells were also increased in CFA-immunized mice. Little is known about the role of γδ T cells in lung disease, but the inflammatory environment of sJIA patients primes blood γδ T cells for IL-17 overproduction (31).

Although all data are in line with the hypothesis of an altered innate immune response in sJIA, the pulmonary manifestations in mice are clearly distinct to those in sJIA patients. At first, the immune cell infiltrates in the lung of CFA-immunized mice were predominantly monocytes, macrophages, and neutrophils, whereas in sJIA patients, mainly lymphoplasmacytic infiltrates were reported. Secondly, different inflammatory pathways are involved in humans and in mice. In humans, IFN-γ and IL-18 play a pivotal role in the activation of resident immune cells in the lungs, including macrophages. This is associated with elevated levels of CXCL9 and CXCL10 in the BAL fluid, which are important in the accumulation of immune cells establishing a self-sustained inflammatory response. Subsequently, this inflammatory response may polarize macrophages away from its surfactant recycling properties, and eventually results in the PAP-like features (17–19). The upregulated IFN-γ signature may also directly promote alveolar macrophage dysfunction since IFN-γ overexpression in T lymphocytes was shown to drive alveolar macrophage dysfunction and PAP-like lung pathology in mice (32). While, both CXCL9 and CXCL10 were upregulated in CFA-immunized WT mice, an IFN-signature was obviously not seen in our CFA-injected IFN-γ KO mice. In our mouse model, the infiltration of neutrophils and monocytes most likely lead to an enhanced production of IL-1β and IL-6 in the lung. Although we did not show direct involvement of these innate immune cells, a positive correlation was reported between the number of neutrophils or Ly6C_pos_ monocytes and the expression levels of, that is, IL-1β. An increased number of myeloid cells was seen in CFA-immunsed IFN-γ KO mice and can be linked to the increased extramedullary myelopoiesis that we previously described in IFN-γ KO mice, challenged with CFA (22, 33), and is also reflected by the increased expression of G-CSF in the lung tissue. Furthermore, in CFA-challenged IFN-γ KO mice, both the IM, the Ly6C_pos_ and the Ly6C_neg_ monocytes/macrophages showed an increased pro-inflammatory activation state, which may possibly sharpen the lung pathology of the sJIA mouse model. Of note, IFN-γ can inhibit the production and/or activity of some pro-inflammatory cytokines or chemokines, including IL-17, TNF-α or GCP-2 however only IL-17 was higher in IFN-γ KO mice compared to WT mice (34–36).

Pathologically, sJIA patients showed ILD with mixed features of PAP and ELP. In our mouse model, we have no evidence of PAP-like features. The absence of PAP-like features may be attributed to differences between mice and humans. Alternatively, the systemic inflammation elicited by CFA as measured in our mouse model, might be insufficient to induce PAP-like lung pathology. In contrast, granulomas were formed in the lungs of CFA-immunized mice. These granulomas were an isolated phenomenon, restricted to the pulmonary environment and were absent in other organs including the liver, heart, kidneys, colon, and brain (data not shown). It indicates that lung expose to pathogen-associated molecular patterns or damage-associated molecular patterns in a systemic inflammatory setting may induce the formation of these granulomas. Note that larger granulomas in CFA-immunized IFN-γ KO mice may be related to the protective role that endogenous IFN-γ has on granuloma formation elicited with killed or live mycobacteria (37–41). No fibrosis was observed in the lung of CFA-immunized IFN-γ mice. This might also be attributed to the absence of IFN-γ, a cytokine that is required in development of bleomycin-induced pneumopathy and fibrosis (42, 43). Of note, fibrosis was also not seen in CFA-challenged WT mice despite the fact that there is evidence of pulmonary inflammation (granuloma formation, infiltration of γδ T cells and a tendency for increased macrophage infiltration). It could also be that the development of fibrosis takes more time than 21 days, the time when our mice were euthanized. Due to ethical reasons assessment of lung disease at later time points was not possible.

A fourth important point of difference between mice and humans, is the use of biologics. In patients, the clinical phenotype and severity of lung disease, coincidend with the introduction of IL-1 and IL-6-targetting biologics (and decreasing used of corticosteroids), suggesting potential contribution of drug exposure (17, 18). In this study, no biologics were used. The exact role of cytokine depletion on lung pathophysiology requires further research.

Pulmonary manifestations are described in several other autoimmune and auto-inflammatory diseases including, that is, Adult-onset Still's disease (AOSD) – which is the adult spectrum of sJIA, rheumatoid arthritis (RA), systemic sclerosis and systemic lupus erythematosus, and are in part associated to the systemic nature of these diseases and the nature of the lungs that are especially vulnerable to a fast innate immune activation (44–46). In AOSD, PLD is reported in 2–12% of the patients. Two main kinds of PLD may be distinguished during AOSD and are either associated with acute respiratory distress syndrome (ARDS) or with another form. The later is characterized by a predominant airway involvement (bronchiolitis and bronchitis) or ILD. Interestingly about half of the cases of non-ARDS PLD was neutrophilic. However, in contrast to our mouse model, no monocyte or macrophage infiltration was reported neither the formation of granulomas. In contrast to sJIA, the pathogenesis of ILD in AOSD has not been clarified yet. Hypercytokinemia with activation of inflammatory cytokines such as IL-6 are suggested to play a crucial role in the pathogenesis (16, 47–49). Note that pulmonary granulomas can be formed in a wide spectrum of pathologies including non-infectious inflammatory sarcoidosis and RA. In RA, these are called rheumatoid nodules (50).

In conclusion, we provided a detailed histologic, immunologic and functional characterization of the pulmonary manifestations in a mouse model of sJIA. We demonstrated early μCT alterations and pulmonary inflammation in both WT and IFN-γ KO BALB/C mice upon CFA-immunization. More extended granuloma formation, cellular infiltrates, cell activation and functional abnormalities were reported in the absence of IFN-γ. Although the pathology of lung inflammation in our model is distinct from human sJIA, it may be representative for a more IL-17-oriented systemic inflammation. Our observations provide evidence that lungs need to be considered as an important target organ in systemic autoinflammation and are a neglected source of pro-inflammatory cytokines that contribute to the cytokine storm. Our study underscore the importance of monitoring lung disease during systemic inflammation and the model provides a tool to explore the underlying mechanism of lung pathology in an autoinflammatory disease context.

## Take-Home Message

Lung complications have recently been described in patients with systemic juvenile idiopathic arthritis (sJIA). In a mouse model for sJIA we characterized the histological, immunological and functional aspects of the lungs.

## Data Availability Statement

The raw data supporting the conclusions of this article will be made available by the authors, without undue reservation.

## Ethics Statement

The animal study was reviewed and approved by Ethics Committee of KU Leuven (P182/2014).

## Author Contributions

BM-D, TD, KD, AV, KA, FP, TM, and LS performed experiments and analyzed data under supervision of PM, JV, GV, CW, and EV. BM-D, TD, PM, JV, and GV were involved in study conceptualization and design. BM-D wrote the initial manuscript, which was critically revised by all other authors. All authors approved the final version of the manuscript.

## Conflict of Interest

The authors declare that the research was conducted in the absence of any commercial or financial relationships that could be constructed as a potential conflict of interest.

## References

[1] Woo P. Systemic juvenile idiopathic arthritis: diagnosis, management, and outcome. Nat Clin Pract Rheumatol. (2006) 2:28–34. 10.1038/ncprheum0084 16932649

[2] Mellins ED Macaubas C Grom AA. Pathogenesis of systemic juvenile idiopathic arthritis : some answers, more questions. Nat Publ Gr. (2011) 7:416–26. 10.1038/nrrheum.2011.68 21647204 PMC4180659

[3] Petty RE Southwood TR Manners P Baum J Glass DN He X. International league of associations for rheumatology classification of juvenile idiopathic arthritis : second revision, edmonton, 2001. J Rheumatol. (2004) 31:390–2. 14760812

[4] Prakken B Albani S Martini A. Juvenile idiopathic arthritis. Lancet. (2011) 377:2138–49. 10.1016/S0140-6736(11)60244-4 21684384

[5] Kessel C Holzinger D Foell D. Phagocyte-derived S100 proteins in autoinflammation: putative role in pathogenesis and usefulness as biomarkers. Clin Immunol. (2013) 147:229–41. 10.1016/j.clim.2012.11.008 23269200

[6] Avau A Put K Wouters CH Matthys P. Cytokine balance and cytokine-driven natural killer cell dysfunction in systemic juvenile idiopathic arthritis. Cytokine Growth Factor Rev. (2015) 26:35–45. 10.1016/j.cytogfr.2014.05.005 24948570

[7] Grom AA Mellins ED. Macrophage activation syndrome : advances towards understanding pathogenesis. Curr Opin Rheumatol. (2010) 22:561–6. 10.1097/01.bor.0000381996.69261.71 20517154 PMC4443835

[8] Macaubas C Nguyen K Deshpande C Phillips C Peck A Lee T. Distribution of circulating cells in systemic juvenile idiopathic arthritis across disease activity states. Clin Immunol. (2010) 134:206–16. 10.1016/j.clim.2009.09.010 19879195 PMC2818241

[9] Ravelli A Davì S Cron R. Macrophage activation syndrome. Hematol Oncol Clin N Am. (2015) 29:927–41. 10.1016/j.hoc.2015.06.010 26461152

[10] Prencipe G Bracaglia C Benedetti F De. Interleukin-18 in pediatric rheumatic diseases. Curr Opin Rheumatol. (2019) 31:421–7. 10.1097/BOR.0000000000000634 31192813

[11] Pascual V Allantaz F Arce E Punaro M Banchereau J. Role of interleukin-1 (IL-1) in the pathogenesis of systemic onset juvenile idiopathic arthritis and clinical response to IL-1 blockade. J Exp Med. (2005) 201:1479–86. 10.1084/jem.20050473 15851489 PMC2213182

[12] Shenoi S. Update on the management of systemic juvenile idiopathic arthritis and role of IL-1 and IL-6 inhibition. Adolesc Health Med Ther. (2017) 8:125–35. 10.2147/AHMT.S109495 29184458 PMC5687245

[13] Ruperto N Brunner HI Quartier P Wulffraat N Horneff G Brik R. Two Randomized trials of canakinumab in systemic juvenile idiopathic arthritis. N engl J Med. (2012) 367:2396–406. 10.1056/NEJMoa1205099 23252526

[14] Yokota S Imagawa T Mori M Miyamae T Aihara Y Takei S. Effi cacy and safety of tocilizumab in patients with systemic-onset juvenile idiopathic arthritis : a randomised, double-blind, placebo-controlled, withdrawal phase III trial. Lancet. (2008) 371:998–1006. 10.1016/S0140-6736(08)60454-7 18358927

[15] Schultz È Mattila J Gappa M Verronen P. Case reports development of progressive pulmonary interstitial and intra-alveolar cholesterol granulomas (PICG) associated with therapy-resistant chronic systemic juvenile arthritis (CJA). Pediatr Pulmonol. (2001) 402:397–402. 10.1002/ppul.1149 11596165

[16] Athreya BH Doughty RA Bookspan M Schumacher HR Sewell EM CJ. Pulmonary manifestations of juvenile rheumatoid arthritis. a report of eight cases and review. Clin Chest Med. (1980) 1:361–74. 7028379

[17] Kimura Y Weiss JE Haroldson KL Lee T Punaro M Oliveira S. Pulmonary hypertension and other potentially fatal pulmonary complications in systemic juvenile idiopathic arthritis. Arthritis Care Res. (2013) 65:745–52. 10.1002/acr.21889 23139240 PMC4476507

[18] Schulert GS Yasin S Carey B Chalk C Schapiro AH Ms AH. Systemic juvenile idiopathic arthritis-lung disease: characterization and risk factors. Arthritis Rheumatol. (2019) 71:1943–54. 10.1002/art.41073 31379071 PMC6817389

[19] Saper VE Chen G Deutsch GH Guillerman RP Birgmeier J Jagadeesh K. Emergent high fatality lung disease in systemic juvenile arthritis. Ann Rheum Dis. (2019) 78:1722–31. 10.1136/annrheumdis-2019-216040 31562126 PMC7065839

[20] Nigrovic PA. Storm warning: lung disease in systemic juvenile idiopathic arthritis. Arthritis Rheumatol. (2019) 71:1773–5. 10.1002/art.41071 31390168 PMC6817387

[21] Klotsche J Raab A Niewerth M Sengler C Ganser G Kallinich T. Outcome and trends in treatment of systemic juvenile idiopathic arthritis in the german national pediatric rheumatologic database, 2000 – 2013. Arthritis Rheumatol. (2016) 68:3023–34. 10.1002/art.39796 27332999

[22] Avau A Mitera T Put K Brisse E Filtjens J Uyttenhove C. Systemic juvenile idiopathic arthritis – like syndrome in mice following stimulation of the immune system with freund' s complete adjuvant. Arthritis Rheumatol. (2014) 66:1340–51. 10.1002/art.38359 24470407

[23] De Jager W Vastert SJ Beekman JM Wulffraat NM Kuis W Coffer PJ. Defective phosphorylation of interleukin-18 receptor b causes impaired natural killer cell function in systemic-onset juvenile idiopathic arthritis. Arthritis Rheum. (2009) 60:2782–93. 10.1002/art.24750 19714583

[24] Put K Vandenhaute J Avau A van Nieuwenhuijze A Brisse E Dierckx T. Inflammatory gene expression profile and defective interferon-γ and granzyme k in natural killer cells from systemic juvenile idiopathic arthritis patients. Arthritis Rheumatol. (2017) 69:213–24. 10.1002/art.39933 27696741

[25] Vande Velde G Poelmans J De Langhe E Hillen A Vanoirbeek J Himmelreich U. Longitudinal micro-CT provides biomarkers of lung disease that can be used to assess the effect of therapy in preclinical mouse models, and reveal compensatory changes in lung volume. Dis Model Mech. (2016) 9:91–8. 10.1242/dmm.020321 26563390 PMC4728330

[26] Berghen N Dekoster K Marien E Dabin J Hillen A Wouters J. Radiosafe micro-computed tomography for longitudinal evaluation of murine disease models. Sci Rep. (2019) 9:1–10. 10.1038/s41598-019-53876-x 31772203 PMC6879529

[27] Vande Velde G De Langhe E Poelmans J Dresselaers T Lories RJ Himmelreich U. Magnetic resonance imaging for noninvasive assessment of lung fibrosis onset and progression cross-validation and comparison of different magnetic resonance imaging bleomycin-induced mouse model. Invest Radiol. (2014) 49:691–8. 10.1097/RLI.0000000000000071 24872004

[28] Devos FC Maaske A Robichaud A Pollaris L Seys S Lopez CA. Forced expiration measurements in mouse models of obstructive and restrictive lung diseases. Respir Res. (2017) 18:123. 10.1186/s12931-017-0610-1 28629359 PMC5477381

[29] Misharin AV Morales-Nebreda L Mutlu GM Budinger GRS Perlman H. Major technical advances flow cytometric analysis of macrophages and dendritic cell subsets in the mouse lung. Am J Respir Cell Mol Biol. (2013) 49:503–10. 10.1165/rcmb.2013-0086MA 23672262 PMC3824047

[30] Tarique AA Logan J Thomas E Holt PG Sly PD Fantino E. Phenotypic, functional, and plasticity features of classical and alternatively activated human macrophages. Am J Respir Cell Mol Biol. (2015) 53:676–88. 10.1165/rcmb.2015-0012OC 25870903

[31] Kessel C Lippitz K Weinhage T Hinze C Wittkowski H Holzinger D. Proinflammatory cytokine environments can drive interleukin-17 overexpression by γ/δ T cells in systemic juvenile idiopathic arthritis. Arthritis Rheumatol. (2017) 69:1480–94. 10.1002/art.40099 28296284

[32] Iriguchi S Kikuchi N Kaneko S Noguchi E Morishima Y Matsuyama M. Regular article T-cell – restricted T-bet overexpression induces aberrant hematopoiesis of myeloid cells and impairs function of macrophages in the lung. Blood. (2015) 125:370–82. 10.1182/blood-2014-05-575225 25349175 PMC4300389

[33] Matthys P Vermeire K Mitera T Huang S Schols D De Wolf-Peeters C. Enhanced autoimmune arthritis in IFN-γ receptor-deficient mice Is conditioned by Mycobacteria in Freund's adjuvant and by increased expansion of Mac-11 myeloid cells. J Immunol. (1999) 163:3505–10. 10477624

[34] Cruz A Khader SA Torrado E Fraga A Pearl JE Pedrosa J. Cutting Edge: IFN- γ Regulates the Induction and Expansion of IL-17-Producing CD4 T Cells during Mycobacterial Infection. J Immunol. (2006) 177:1416–20. 10.4049/jimmunol.177.3.1416 16849446

[35] Chu C Swart D Alcorn D Tocker J Elkon KB Carson L. Interferon-g regulates susceptibility to collagen-induced arthritis through suppression of interleukin-17. Arthritis Rheum. (2007) 56:1145–51. 10.1002/art.22453 17393396

[36] Kelchtermans H Struyf S Klerck B De Mitera T Alen M Geboes L. Protective role of IFN-g in collagen-induced arthritis conferred by inhibition of mycobacteria-induced granulocyte chemotactic protein-2 production. J Leukoc Biol. (2006) 81:1044–53. 10.1189/jlb.0806486 17200147

[37] Tomashefski JF Farver CF. Tuberculosi and nontuberculous mycobacterial infection. In: Tomashefski JF Cagle PT Farver CF Fraire AE, editors. Dail and Hammar's Pulmonary Pathology. New York, NY: Springer (2008) p. 316–48. 10.1007/978-0-387-68792-6_9

[38] Gilbert S Steinbreck DS Landas SK Hunninghake GW. Amounts of angiotensi-converting enzyme mRNA reflect the burden of granulomas in granulomatous lung disease. Am Rev Respir Dis. (1993) 148:483–6. 10.1164/ajrccm/148.2.483 8393640

[39] Schurgers E Mertens F Vanoirbeek JAJ Mitera T Langhe E De Billiau A. Pulmonary inflammation in mice with collagen- induced arthritis is conditioned by complete Freund' s adjuvant and regulated by endogenous IFN- γ. Eur J Immunol. (2012) 3223–34. 10.1002/eji.201242573 22930199

[40] Kamijo R Le J Shapiro DA Edward H Huang SS Aguet M. Mice that lack the interferon-g receptor have profoundly altered responses to infection with bacillus calmette-guérin and subsequent challenge with lipopolysaccharide. J Exp Med. (1993) 178:1435–40. 10.1084/jem.178.4.1435 8376946 PMC2191201

[41] Flynn BJL Chan J Triebold KJ Dalton DK Stewart TA Bloom BR. An essential role for interferon-g in resistance to mycobacterium tuberculosis infection. J Exp Med. (1993) 178:2249–54. 10.1084/jem.178.6.2249 7504064 PMC2191274

[42] Chen ES Greenlee BM Wills-karp M Moller DR. Attenuation of lung inflammation and fibrosis in interferon-g-deficient mice after intratracheal bleomycin. Am J Respir Cell Mol Biol. (2001) 24:545–55. 10.1165/ajrcmb.24.5.4064 11350823

[43] Ulloa L Doody J Massagué J. Inhibition of transforming growth factor-b/SMAD signalling by the interferon-g/STATpathway. Nature. (1999) 601:710–3. 10.1038/17826 10067896

[44] Alamoudi OSB Attar SM. Pulmonary manifestations in systemic lupus erythematosus: association with disease activity. Respirology. (2015) 20:474–80. 10.1111/resp.12473 25639532 PMC4418345

[45] Solomon JJ Olson AL Fischer A Bull T Brown KK Raghu G. Scleroderma lung disease. Eur Respir J. (2013) 22:6–19. 10.1183/09059180.00005512 PMC4103193 23457159

[46] Yunt ZX Solomon JJ. Lung disease in rheumatoid arthritis. Rheum Dis Clin North Am. (2015) 41:225–36. 10.1016/j.rdc.2014.12.004 25836639 PMC4415514

[47] Gerfaud-valentin M Cottin V Jamilloux Y Hot A Gaillard-coadon A Durieu I. Parenchymal lung involvement in adult-onset still disease. Medicine. (2016) 95:1–10. 10.1097/MD.0000000000004258 27472698 PMC5265835

[48] Guerrieri A Angeletti G Mazzolini M Bassi I Nava S. Respiratory medicine case reports pulmonary involvement in adult still' s disease : case report and brief review of literature. Respir Med Case Reports. (2017) 22:91–4. 10.1016/j.rmcr.2017.07.001 PMC5503835 28725546

[49] Takakuwa Y Hanaoka H Kiyokawa T Iida H Ishimori K Uekusa T. Adult-onset Still' s disease-associated interstitial lung disease represents severe phenotype of the disease with higher rate of haemophagocytic syndrome and relapse. Clin Exp Rheumatol. (2019) 27:S23–7. 30767871

[50] Ohshimo S Guzman J Costabel U Bonella F. Differential diagnosis of granulomatous lung disease : clues and pitfalls. Eur Respir J. (2017) 26:170012. 10.1183/16000617.0012-2017 28794143 PMC9488688

