# Supplementary material for: Lung Functioning and Inflammation in a Mouse Model of Systemic Juvenile Idiopathic Arthritis
Source: Front Immunol. 2021 Mar 12;12:642778. doi: 10.3389/fimmu.2021.642778 (PMC7996094; doi:10.3389/fimmu.2021.642778)
Supplement: Supplementary file 1 [file Data_Sheet_1.pdf]

Lung functioning and inflammation in a mouse model of systemic juvenile idiopathic arthritis

Bert Malengier-Devlies[1], Tatjana Decaesteker[2], Kaat Dekoster[3], Arno Vanstapel[2], Kourosh Ahmadzadeh[1], Fariba Poosti[4], Tania Mitera[1], Laura Seldeslachts[3], Erik Verbeken[5], Carine Wouters[1,6,7], Greetje Vande Velde[3], Jeroen Vanoirbeek[2], Patrick Matthys[1]

[1] Laboratory of Immunobiology, Department of Microbiology and Immunology, KU Leuven, Leuven, Belgium
[2] Laboratory of Respiratory Diseases and Thoracic Surgery (BREATHE), Department of Chronic Diseases and Metabolism, KU Leuven, Leuven, Belgium
[3] Biomedical MRI, Department of Imaging & Pathology, KU Leuven, Leuven, Belgium
[4] Laboratory of Molecular Immunology, Department of Microbiology and Immunology, KU Leuven, Leuven, Belgium
[5] Morphology and Molecular Pathology Section, University Hospitals Leuven, Leuven, Belgium
[6] Division of Pediatric Rheumatology, University Hospitals Leuven, Leuven, Belgium
[7] European Reference Network for Rare Immunodeficiency, Autoinflammatory and Autoimmune Diseases (RITA) at University Hospital Leuven, Leuven, Belgium

Address for correspondence:
Patrick Matthys, Laboratory of Immunobiology, Rega Institute, KU Leuven, Herestraat 49 box 1044, 3000 Leuven, Belgium
Tel: +32 16 32 23 63
Email: patrick.matthys@kuleuven.be

Supplementary Tables

Supplementary Table 1

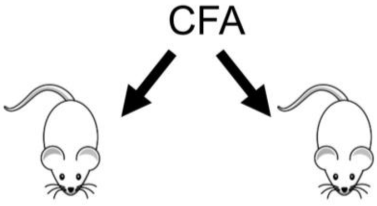

**sJIA characteristics**	WT	IFN-γ KO
Clinical features		
- Fever	-	-
- Weight loss	+	++
- Arthritis	-	+[a]
- Skin rash	-	+[a]
- Splenomegaly	+	++
- Lymphadenopathy	+	+
Hematologic features		
- Neutrophilia	+	++
- Thrombocytosis	+	++
- Lymphocytopenia	-	+
- Anaemia	-	+[a]
Immune/inflammatory features		
- Increased IL-6	-	+
- Decreased NK cell functionality	-	+

Features were graded as follows: +, present;, ++, more pronounced present; and -, absent.
[a] In subsets of mice.
For WT and IFN-γ KO mice, a summary of more than 10 experiments was used to compose this table, and the list of features is founded on data published in Avau et al., Imbrechts et al. and Vandenhaute et al. (4–6)

Supplementary Table 2

Antigen	Fluorophore	Clone	Company
B220	BV786	RA3-6B2	BD
CD101	Af647	307707	BD
CD103	PerCpEF710	2E7	eBioscience
CD115	PE	AFS98	eBioscience
CD117	PE-Cy5	2B8	Biolegened
CD11b	BUV395	M1/70	BD
CD11b	BUV395	M1/70	BD
CD11c	BV711	N418	Biolegend
CD11c	BV711	N418	Biolegend
CD122	BV421	TM-b1	BD
CD16/32	APC-Cy7	93	Biolegend
CD163	APC	S150491	Biolegend
CD19	BV510	6D5	Biolegend
CD19	FITC	eBio1D3	eBioscience
CD206	BV605	C068C2	Biolegend
CD27	FITC	LG.7G9	eBioscience
CD3	BV510	17A2	Biolegend
CD3	FITC	145-2C11	eBioscience
CD3	BUV737	145-2C11	BD
CD34	FITC	RAM34	BD
CD4	PerCp-Cy5.5	RM4-5	eBioscience
CD4	APC-ef780	RM4.5	eBioscience
CD45	PE	30-F11	eBioscience
CD64	BV786	X54-5/7.1	BD
CD8	BV711	53-6.7	BD
CD80	BV421	16-1OA1	BD
CXCR2	PerCp-Cy5.5	SA045E1	Biolegend
CXCR4	BV605	L276F12	Biolegend
DX5	FITC	DX5	eBioscience
DX5	PE-Cy5	DX5	eBioscience
IL-17	eF450	eBio1787	eBioscience
IL-1β	PE	REA577	Milteny Biotec
IL-6	FITC	MP5-20F3	eBioscience
Ly6C	APC-Cy7	AL-21	BD
Ly6C	PerCp-Cy5.5	HK1.4	eBioscience
Ly6G	BV786	1A8	BD
Ly6G	FITC	1A8-LY6G	eBioscience
Ly6G	BV786	1A8	BD
MHCII	AlexaFluor700	M5/114.14.2	eBioscience
SiglecF	PE-CF594	E50-2440	BD
TNF-α	APC	MP6-XT22	eBioscience
ΓδTCR	APC	GL3	Biolegend
ΓδTCR	PE-Cy7	GL3	Biolegend

Supplementary Table 3

Gene	Primer name	Company
IL1β	Mm.PT.58.42940223	IDT
IL-6	Mm. PT.58.10005566	IDT
TNF-α	Mm.PT.58.1257581	IDT
IL-17	Mm.PT.58.6531092	IDT
IL-22	Mm00444241_m1	Thermo Fisher
IL-10	Mm.PT.58.13531087	IDT
KC	Mm.PT.58.42076891	IDT
MIP2	Mm.PT.58.10456839	IDT
GCP2	Mm00436451_g1	Thermo Fisher
G-CSF	Mm.PT58.7976206.gs	IDT
MCP1	Mm.PT.58.42151692	IDT
GAPDH	Mm.PT.39a.1	IDT
IFNA1	Mm.PT.58.43426930.g	IDT
IFNB1	Mm.PT.58.30132453.g	IDT
CXCL9	Mm.PT.58.5726745	IDT
CXCL10	Mm.PT.58.43575827	IDT
IFIT1	Mm.PT.58.32674307	IDT
ISG15	Mm.PT.58.41476392.g	IDT
SIGLEC1	Mm.PT.58.8446763	IDT
