# Supplementary figures and images for: Lung Functioning and Inflammation in a Mouse Model of Systemic Juvenile Idiopathic Arthritis
Source: Front Immunol. 2021 Mar 12;12:642778. doi: 10.3389/fimmu.2021.642778 (PMC7996094; doi:10.3389/fimmu.2021.642778)

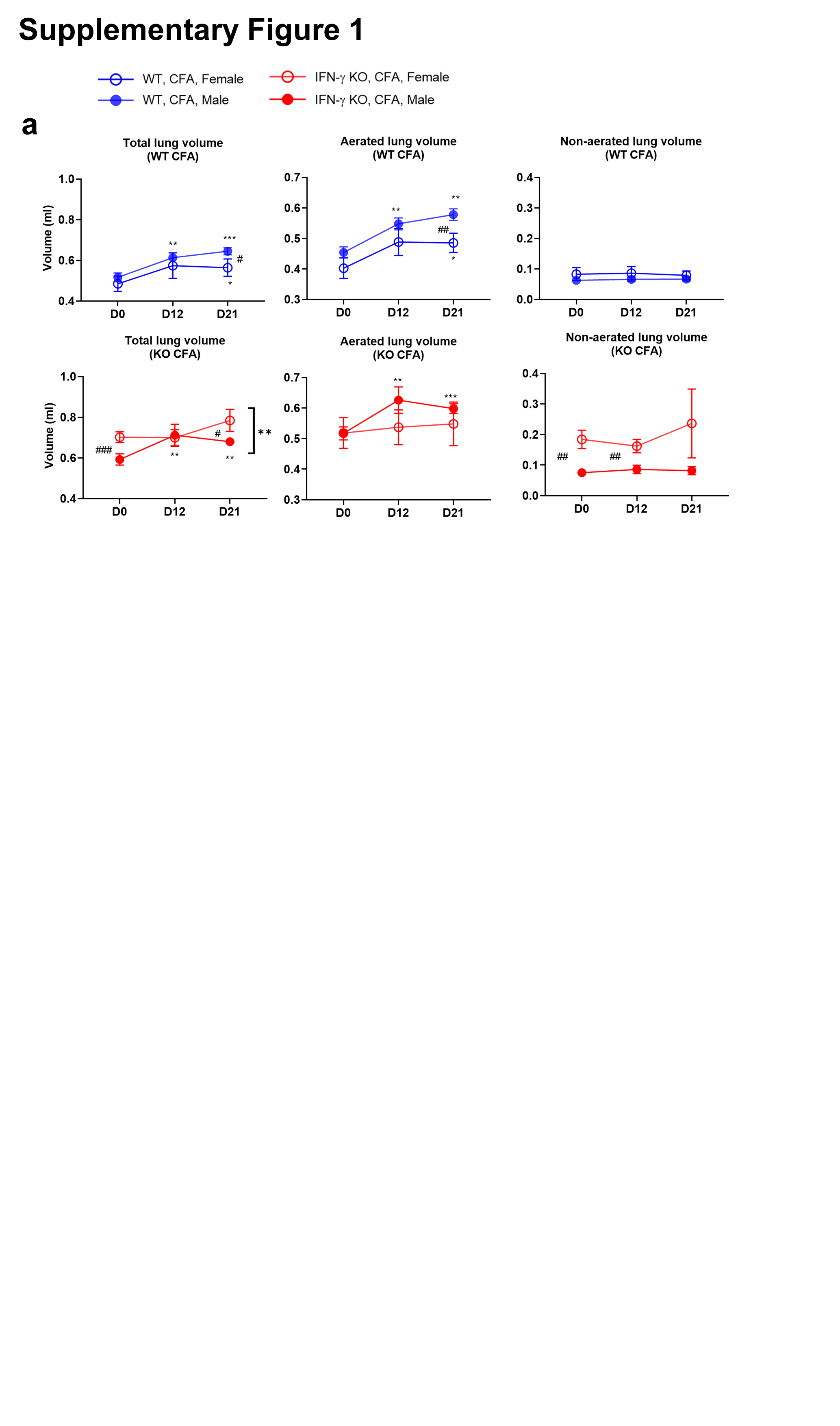

Supplement: Supplementary file 2 [file Data_Sheet_2.zip › Supplementary Figure 1.TIF]

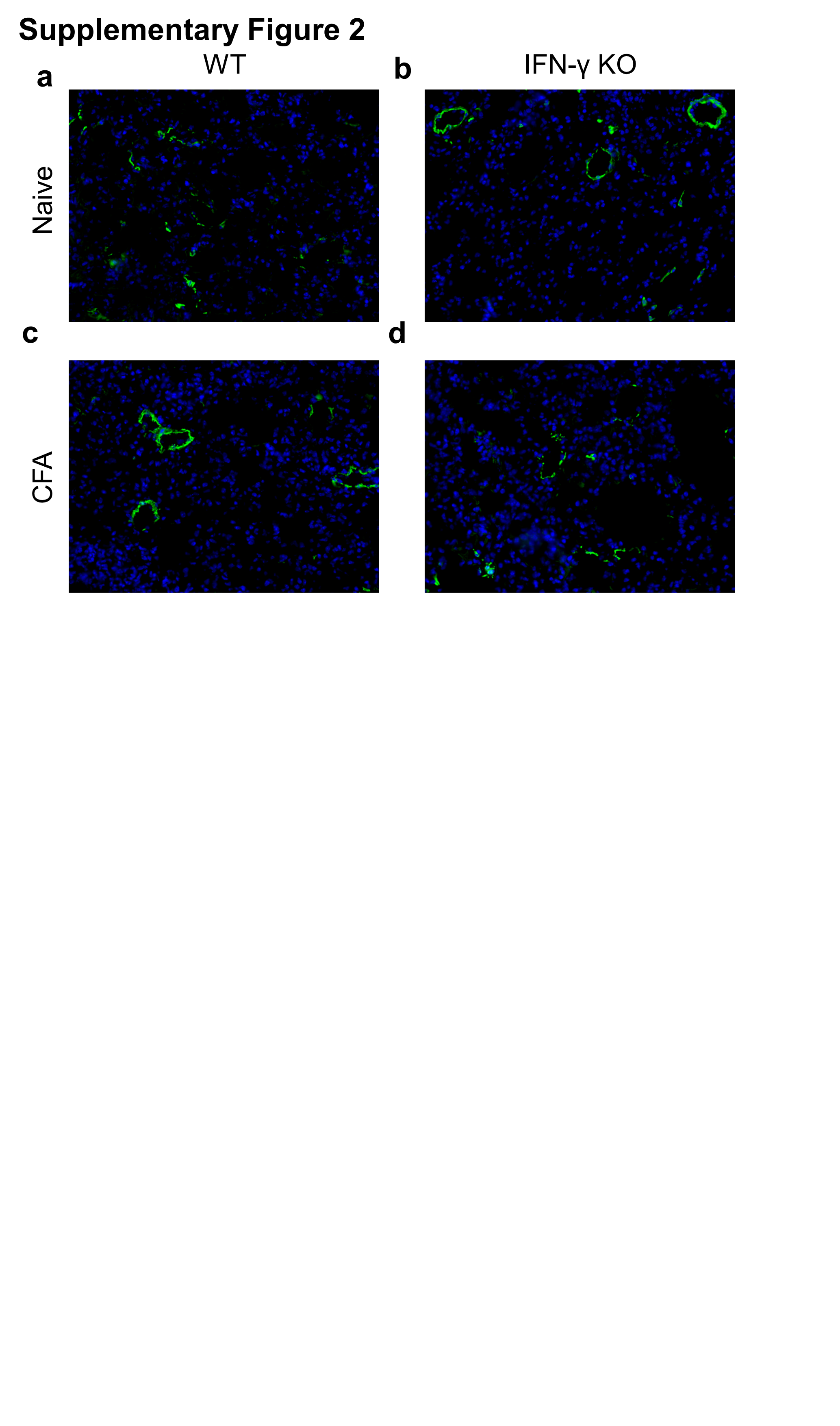

Supplement: Supplementary file 2 [file Data_Sheet_2.zip › Supplementary Figure 2.TIF]

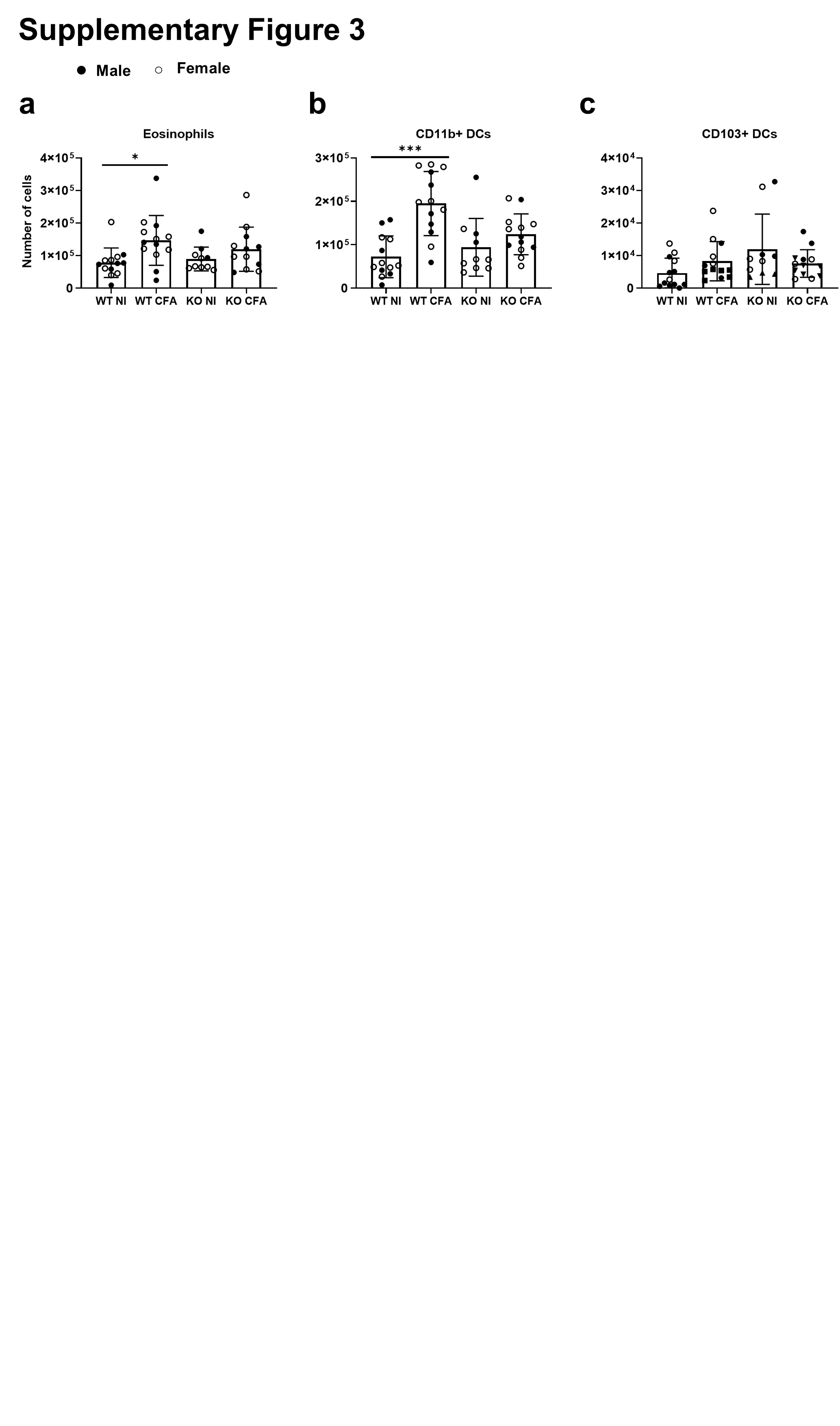

Supplement: Supplementary file 2 [file Data_Sheet_2.zip › Supplementary Figure 3.TIF]

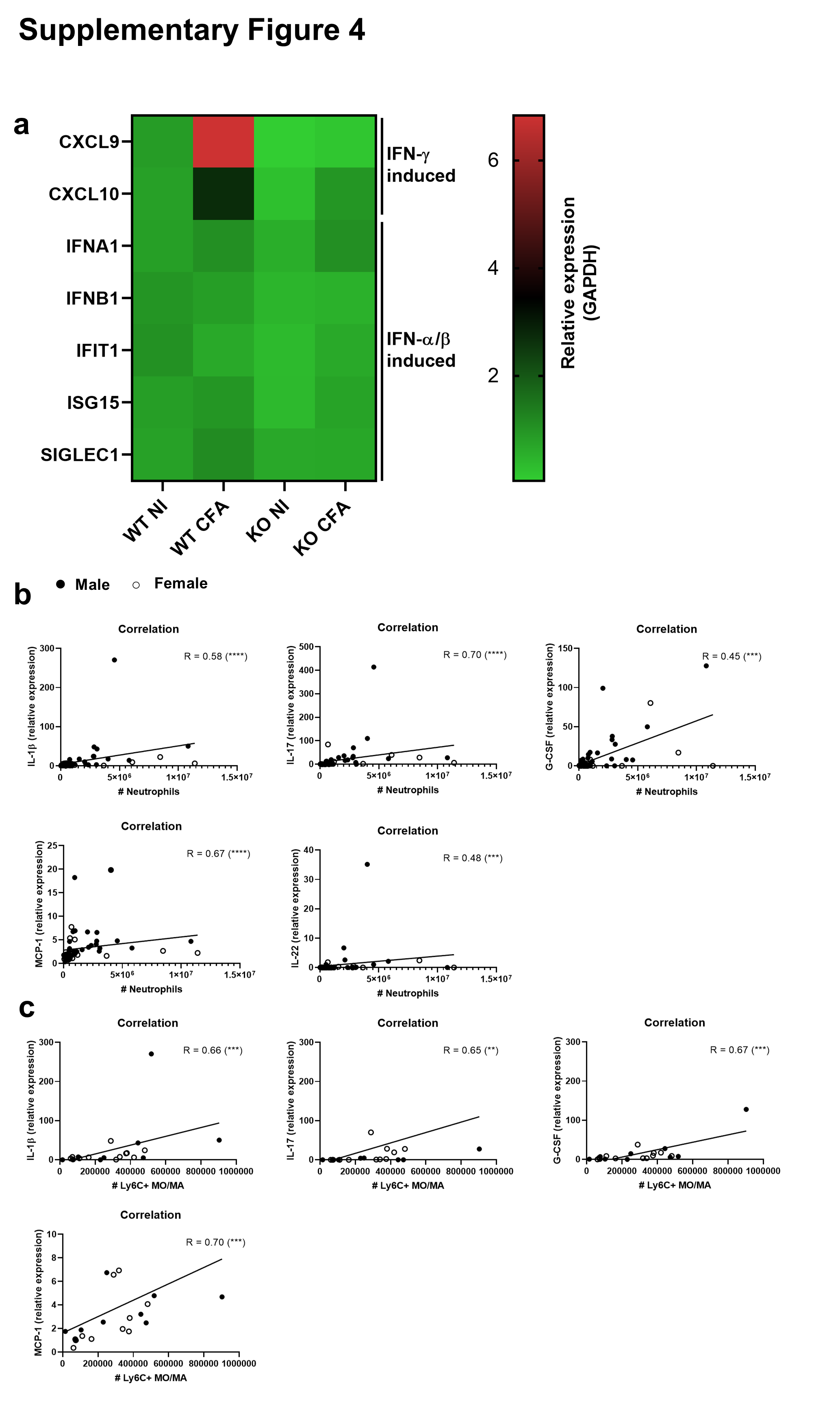

Supplement: Supplementary file 2 [file Data_Sheet_2.zip › Supplementary Figure 4.TIF]
